# The number of nuclei in compacted embryos, assessed by optical coherence microscopy, is a non-invasive and robust marker of mouse embryo quality

**DOI:** 10.1093/molehr/gaae012

**Published:** 2024-02-24

**Authors:** Aleksandra Sobkowiak, Monika Fluks, Ewa Kosyl, Robert Milewski, Marcin Szpila, Szymon Tamborski, Maciej Szkulmowski, Anna Ajduk

**Affiliations:** Department of Embryology, Institute of Developmental Biology and Biomedical Sciences, Faculty of Biology, University of Warsaw, Warsaw, Poland; Department of Embryology, Institute of Developmental Biology and Biomedical Sciences, Faculty of Biology, University of Warsaw, Warsaw, Poland; Department of Embryology, Institute of Developmental Biology and Biomedical Sciences, Faculty of Biology, University of Warsaw, Warsaw, Poland; Department of Biostatistics and Medical Informatics, Medical University of Bialystok, Białystok, Poland; Department of Embryology, Institute of Developmental Biology and Biomedical Sciences, Faculty of Biology, University of Warsaw, Warsaw, Poland; Department of Biophotonics and Optical Engineering, Institute of Physics, Faculty of Physics, Astronomy, and Informatics, Nicolaus Copernicus University in Torun, Toruń, Poland; Department of Biophotonics and Optical Engineering, Institute of Physics, Faculty of Physics, Astronomy, and Informatics, Nicolaus Copernicus University in Torun, Toruń, Poland; Department of Embryology, Institute of Developmental Biology and Biomedical Sciences, Faculty of Biology, University of Warsaw, Warsaw, Poland

**Keywords:** embryo quality, embryo development, compacted embryo, morula, blastocyst, implantation, optical coherence microscopy, animal model

## Abstract

Optical coherence microscopy (OCM) visualizes nuclei in live, unlabeled cells. As most cells are uninucleated, the number of nuclei in embryos may serve as a proxy of the cell number, providing important information on developmental status of the embryo. Importantly, no other non-invasive method currently allows for the cell number count in compacted embryos. We addressed the question of whether OCM, by providing the number of nuclei in compacted mouse embryos, may help evaluate embryo quality. We subjected compacted embryonic Day 3 (E3.0: 72 h after onset of insemination) mouse embryos to OCM scanning and correlated nuclei number and developmental potential. Implantation was assessed using an outgrowth assay (*in vitro* model meant to reflect embryonic ability to implant *in vivo*). Embryos with more cells at E3.0 (>18 cells) were more likely to reach the blastocyst stage by E4.0 and E5.0 (*P* ≪ 0.001) and initiate hatching by E5.0 (*P* < 0.05) than those with fewer cells (<12 cells). Moreover, the number of cells at E3.0 strongly correlated with the total number of cells in E4.0 and E5.0 embryos (*ρ* = 0.71, *P* ≪ 0.001 and *ρ* = 0.61, *P* ≪ 0.001, respectively), also when only E4.0 and E5.0 blastocysts were considered (*ρ* = 0.58, *P* ≪ 0.001 and *ρ* = 0.56, *P* ≪ 0.001, respectively). Additionally, we observed a strong correlation between the number of cells at E3.0 and the number of trophectoderm cells in E4.0 and E5.0 blastocysts (*ρ* = 0.59, *P* ≪ 0.001 and *ρ* = 0.57, *P* ≪ 0.001, respectively). Importantly, embryos that had more cells at E3.0 (>18 cells) were also more likely to implant *in vitro* than their counterparts with fewer cells (<12 cells; *P* ≪ 0.001). Finally, we tested the safety of OCM imaging, demonstrating that OCM scanning affected neither the amount of reactive oxygen species nor mitochondrial activity in the embryos. OCM also did not hinder their preimplantation development, ability to implant *in vitro*, or to develop to term after transfer to recipient females. Our data indicate that OCM imaging provides important information on embryo quality. As the method seems to be safe for embryos, it could be a valuable addition to the current repertoire of embryo evaluation methods. However, our study was conducted only on mouse embryos, so the proposed protocol would require optimization in order to be applied in other species.

## Introduction

Quality assessment of embryos is one of the key aspects of the IVF procedure. If conducted properly, it may optimize the IVF success rate, especially when only a single embryo can be transferred to the female uterus. Therefore, infertility clinics employ an array of methods meant to evaluate the developmental potential of embryos. The standard protocol for evaluating human embryo quality involves assessment of morphology on subsequent days of *in vitro* culture up to the blastocyst stage. Zygotes are usually graded according to the morphology of their pronuclei and cytoplasm. Healthy cleaving embryos have evenly sized, uninuclear blastomeres with no or limited fragmentation. High-quality blastocysts have a distinct blastocoele, a trophectoderm (TE) layer (participating in embryo implantation and placentation), and an inner cell mass (ICM; giving rise to the future fetus body and fetal membranes) (Ebner *et al.*, 2003; Ajduk and Zernicka-Goetz, 2013; Anagnostopoulou *et al.*, 2022). Nowadays, static visual assessment is often replaced by time-lapse imaging of embryos, a method that allows for a more detailed analysis of embryonic morphology (particularly its transient stages, such as reverse or direct cleavage divisions, and fragmentation) and rate of preimplantation development (Montag *et al.*, 2013; Kovacs, 2014; Milewski and Ajduk, 2017; Gallego *et al.*, 2019).

However, both static and time-lapse-based assessment of embryonic morphology become difficult when embryos undergo compaction, i.e. their cells flatten against each other, increasing the contact areas and making the embryo more spherical. In mouse embryos, compaction typically occurs at the late eight-cell stage, whereas in human embryos, it occurs at ∼16-cell stage (reviewed in Płusa and Piliszek, 2020). According to the research carried out primarily on mouse embryos, compaction is driven by a complex mechanism involving E-cadherin-mediated cell adhesion, actomyosin-generated cortical tension, and filopodia (reviewed in White *et al.*, 2016). During this process, the borders between blastomeres become invisible in transmitted light. Therefore, the quality of compacted embryos is currently evaluated mostly based on the completeness of compaction and level of fragmentation (Ebner *et al.*, 2009; Ivec *et al.*, 2011; Fabozzi *et al.*, 2016; Tsai *et al.*, 2019; Coticchio *et al.*, 2021b; Li *et al.*, 2021; Hur *et al.*, 2023). From this stage onwards, the assessment of cell size or cell number by standard light microscopy is impossible. This is unfortunate, as the number of blastomeres in late compacted embryos could be an objective measure of their developmental advancement, especially as the compaction stage precedes two major developmental events: cavitation and implantation. However, recent advances in optical microscopy may provide a solution to this situation.

Optical coherence microscopy (OCM) is a novel, non-invasive, 3D imaging method, capable of imaging at a uniform resolution on a micrometer scale in all spatial directions (Zheng *et al.*, 2012; Raghunathan *et al.*, 2016; Karnowski *et al.*, 2017; Ajduk and Szkulmowski, 2019). It is derived from optical coherence tomography, which is widely applicable in medicine as the primary method for tissue 3D structural and functional imaging *in vivo*. In particular, OCM allows for clear visualization of nuclei in oocytes and embryos (Karnowski *et al.*, 2017; Fluks *et al.*, 2022). Moreover, its invasiveness is negligible, as it utilizes scattered light intensity as the contrasting mechanism and therefore requires no additional staining. We have previously demonstrated that our optimized OCM protocols can shorten the imaging time, decrease the required light intensity, and reduce noise (Karnowski *et al.*, 2017). Recently, we have also shown that OCM can be a useful tool in the quality assessment of immature oocytes (Fluks *et al.*, 2022). To date, OCM has been applied to visualize oocytes and preimplantation embryos of mice, pigs, and cows (Xiao *et al.*, 2012; Zheng *et al*, 2012, 2013; Karnowski *et al.*, 2017; Moore *et al.*, 2019; Masuda *et al.*, 2021; Fluks *et al.*, 2022).

In the present study, we applied a mouse model to examine whether OCM may be used for the quality assessment of compacted embryos. We focused on analyzing the number of nuclei (as a proxy for the cell number) in embryonic Day 3 (E3.0) compacted embryos, i.e. just prior to cavitation. We have chosen this stage instead of fully grown blastocysts because at this time-point, cells are bigger and less numerous than in blastocysts, so their nuclei are easier to visualize and count. Additionally, if the method is going to be introduced to clinical or veterinary practice in the future, conducting OCM scanning earlier in embryo development warrants more time for the necessary data analysis. We investigated whether the number of nuclei in E3.0 compacted embryos correlates with ability of the embryo to cavitate, hatch, and form the first embryonic cell lineages, i.e. TE and ICM-localized epiblast (EPI) and primitive endoderm (PE; or hypoblast). During further embryonic development, EPI cells will give rise to the fetus body, whereas PE and TE cells will form extraembryonic structures, such as the yolk sac and embryonic part of the placenta, respectively (reviewed in Mihajlović and Bruce, 2017). Using an outgrowth assay, i.e. an *in vitro* model meant to reflect the embryonic ability to implant *in vivo* (Kim *et al.*, 2020), we also examined whether the cell number in E3.0 compacted embryos is related to their ability to undergo implantation. Finally, we verified whether OCM imaging is safe for embryos by comparing pre- and postimplantation development of OCM-scanned and non-imaged embryos.

## Materials and methods

### Animals

Mice were maintained in the animal facility of the Faculty of Biology, University of Warsaw under a 14:10 light/darkness cycle and provided with food and water *ad libitum*. Animals were killed by cervical dislocation. All animal experiments were approved by the Local Ethical Committee for Experimentation on Animals No. 1 in Warsaw (Poland; approval nos. 698/2018 and 993/2020) and were performed in compliance with the national regulations (Act on the Protection of Animals Used for Scientific and Educational Purposes from 15 January 2015) and reported according to the ARRIVE guidelines (https://arriveguidelines.org).

### Isolation of *in vivo*-produced compacted embryos

Superovulation of 2- to 4-month-old F1 (C57Bl6/Tar × CBA/Tar) female mice was achieved with an i.p. injection of 7.5 IU of pregnant mare’s serum gonadotrophin (PMSG; BioVendor R&D, Brno, Czech Republic) followed 48 h later by 7.5 IU of hCG (Intervet, Warsaw, Poland). The mice were mated with 6- to 12-month-old F1 males. Seventy-two hours later, embryos were isolated from the oviducts and uteri of the mated females into prewarmed M2 medium (M16 medium buffered with HEPES (Fulton and Whittingham, 1978)). In most experiments, however, embryos were obtained by IVF.

### IVF

Superovulation of 2- to 5-month-old F1 (C57Bl6/Tar × CBA/Tar) female mice was achieved with an i.p. injection of 7.5 IU of PMSG followed 48 h later by 7.5 IU of hCG. Fifteen hours later, oocytes surrounded by cumulus cells were recovered from the oviducts into 100-μl droplets of fertilization medium (Fraser, 1982) with 5 mg/ml bovine serum albumin (BSA, Merck, Poznań, Poland). Fertilization medium was pre-equilibrated with 5% CO_2_ for at least 30 min before use. Epididymal spermatozoa were isolated from 6- to 12-month-old F1 male mice and capacitated in 0.5 ml of fertilization medium for 1.5–2 h, and then 10 μl of sperm suspension was added to the droplets containing oocytes. Gametes were co-incubated under standard culture conditions (37.5°C and 5% CO_2_ in the air) for 4 h and then loose spermatozoa were washed off fertilized oocytes. Fertilized oocytes were moved in groups of 10 to 10-μl droplets of KSOM medium without amino acids (EmbryoMax, Speciality Media, Merck, Poznań, Poland) covered with mineral oil for further culture under standard conditions (37.5°C and 5% CO_2_ in the air). The timing of embryo development was counted from the onset of insemination (i.e. E3.0 means 72 h after the onset of insemination).

### Optical coherence microscopy

**Figure 1.**
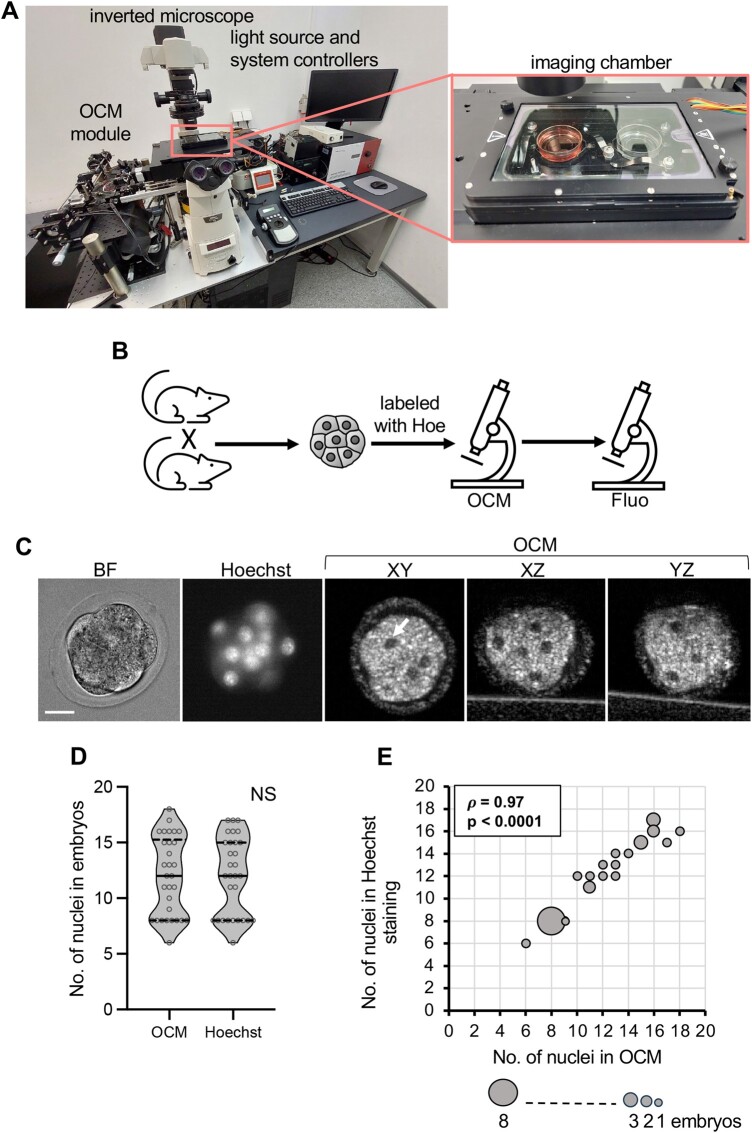
**Verification of the count of nuclei in mouse compacted embryos obtained by optical coherence microscopy**. (**A**) Custom-made optical coherence microscopy (OCM) set-up used in the experiments. The insert shows the imaging chamber. (**B**) Scheme of the experimental design. (**C**) Images of a representative compacted embryo: in bright field, fluorescence microscopy (DNA stained with Hoechst 33342), and in three different planes (XY, XZ, and YZ) in OCM. Scale bar 20 µm. (**D**) Comparison of the number of nuclei in compacted embryos (n = 30) counted according to OCM and fluorescence imaging. Violin plots show distribution of the analyzed data; the solid black line indicates median, the dashed black lines—first and third quartile values; NS: *P* > 0.05. (**E**) Spearman’s rank correlation between numbers of nuclei visualized in compacted embryos (n = 30) by OCM and fluorescence microscopy. The size of the dots reflects the number of represented embryos. BF: bright field; Fluo: fluorescence microscopy; Hoe: DNA dye, Hoechst 33342.

We used the same OCM set-up as in our previous study (Karnowski *et al.*, 2017). In short, a custom-made fiber-based OCM imaging system was combined with an inverted microscope (Eclipse Ti-E, Nikon, Amstelveen, The Netherlands) equipped with a culture chamber maintained at a constant temperature during the experiment (OkoLab, Napoli, Italy) (Fig. 1A). The system was also equipped with a broadband supercontinuum light source (SuperK EXTREME EXW-4 with SuperK Split spectral splitter, NKT Photonics A/S, Birkerød, Denmark) that provided a spectrum as broad as 115 nm (measured at the level of –3 dB intensity drop) and centered at 800 nm, which resulted in axial resolution of 1.9 µm (in depth, *Z*-axis). The OCM interferometer sample arm shared an imaging objective (Nikon Plan Fluor 20x, NA = 0.5) with an inverted microscope system, which provided 1.9 µm transverse resolution (lateral, X–Y axes). The average optical power of the focused probing beam at the sample plane was 1.2 mW. The OCM acquisition was possible with a maximum rate of 140 000 lines of image per second using a line-scan complementary metal oxide semiconductor (CMOS) camera (Basler Sprint spL2048-140 km, Basler AG, Ahrensburg, Germany). The spectra were digitized with a high-bandwidth framegrabber (PCIe-1433, National Instruments, Austin, TX, USA). The sensitivity of the instrument was 89 dB (measured at a typical 20 µs exposure time). The imaging range in depth was 0.9 mm. The OCM probing beam scanned a sample in both lateral directions using a pair of galvanometric scanners (8320 K, Cambridge Technology Inc., Bedford, MA, USA) driven by an analog I/O card (PCI-6733, National Instruments, Austin, TX, USA). The spectra acquired for every point on the X–Y plane were processed to obtain a tomogram line along the *Z*-axis. The processing protocol consisted of fixed pattern noise removal, resampling to the wavenumber domain, residual dispersion correction, spectrum shaping, and Fourier transformation (Szkulmowski *et al.*, 2005). The measurements were controlled by custom-designed software (C++/C#), which assured precise synchronization between all components of the system.

### OCM imaging of mouse embryos

Only compacted embryos were used for OCM imaging. Embryos that at E3.0 were arrested at the 2–8-cell stage and remained uncompacted were excluded from the analysis, as being clearly of poor quality. Compacted embryos were transferred to a glass bottom dish filled with prewarmed M2 medium. The glass bottom was coated with a thin (∼100–150 μm) layer of agar (1% solution in 0.9% NaCl, Bacto-agar, BD, Warsaw, Poland) to avoid the unwanted, strong OCM signal from the glass surface. The medium was covered with a layer of mineral oil (BioXtra, Merck, Poznań, Poland) to prevent evaporation. The dish was then placed on the inverted microscope in a chamber maintained at the constant temperature of 37.5°C. The images were acquired as described previously (Fluks *et al.*, 2022): 15 3D volumes were acquired in five series 12-s apart, each composed of three volumes 3-s apart, and then averaged. Each volume was composed of 250 OCM lines in each lateral direction. OCM images were then used to calculate the number of nuclei (as a proxy for the number of cells) in scanned embryos. Each scan took ∼1 min, embryos were scanned in batches of maximum 10 and were kept on the microscope for ∼30 min. Scanned embryos were then moved individually to 2-µl droplets of KSOM medium (without amino acids) for an additional 24 or 48 h of *in vitro* culture under standard conditions (37.5°C and 5% CO_2_ in the air). KSOM medium was pre-equilibrated with 5% CO_2_ for at least 30 min before use.

In some experiments, OCM-scanned embryos, stained earlier for 30 min with 100 ng/ml Hoechst 33342 (in M2 medium; Merck, Poznań, Poland), were imaged on a fluorescence microscope (Zeiss Axiovert, Jena, Germany) to verify the number of their nuclei.

When the quality of OCM-scanned and control embryos was compared, both groups of embryos were kept in the same conditions during the time required for OCM scanning: in M2 medium under mineral oil, at 37.5°C, and in the atmospheric concentration of CO_2_. The only difference between those groups was that one was subjected to an OCM scan and the other was not.

### Detection of reactive oxygen species and mitochondrial activity

To visualize reactive oxygen species (ROS), embryos were incubated for 30 min at 37.5°C in 5 μM CellROX Orange (Thermo Fisher Scientific, Warsaw, Poland), a fluorescent ROS indicator. A fluorescence microscope (Zeiss Axiovert, Jena, Germany) equipped with an AxioCam HRm camera was used to obtain single-plane images of the embryos’ equatorial region. Embryos were illuminated with light passing through a 538–562 nm excitation filter, and the emitted light was collected with a 570–640 nm emission filter (exposition time 100 ms, 4 × 4 binning). To assess ROS concentration, the mean intensity of CellROX Orange fluorescence was measured. In each experiment, the mean fluorescence intensity in OCM-scanned embryos (F) was normalized with the mean fluorescence intensity in control (unscanned) embryos, which were dyed and imaged simultaneously (F_CTRL_).

To visualize mitochondrial activity, embryos were incubated for 30 min at 37.5°C in 2 μM JC-1 (Thermo Fisher Scientific, Warsaw, Poland). JC-1 is a cationic indicator of mitochondrial activity, which at low concentrations is monomeric and emits green light, while at high concentrations forms aggregates that emit red light. A fluorescence microscope (Zeiss Axiovert, Jena, Germany) equipped with an AxioCam HRm camera was used to acquire stacks of 10 images along the embryo’s *Z*-axis (5.5 μm apart). Embryos were illuminated with light passing through 450–490 nm and 528–562 nm excitation filters and the emitted light was collected using 500–550 nm and 570–640 nm emission filters (exposition time of 20 ms for green and 15 ms for red channels, 4 × 4 binning). To assess mitochondrial activity, a ratio of red to green JC-1 fluorescence measured for the sum of all 10 stack images was calculated. In each experiment, the ratio in each OCM-scanned embryo was normalized with the mean ratio in control (unscanned) embryos, which were dyed and imaged simultaneously.

### Immunofluorescence staining

At E4.0 or E5.0, embryos were fixed individually in 4% paraformaldehyde (Merck, Poznań, Poland; 30 min at room temperature), permeabilized with 0.5% Triton X-100 (Merck, Poznań, Poland; 30 min at room temperature), and blocked with 3% BSA (overnight, 4°C). CDX2, a TE marker, was labeled with mouse monoclonal antibody (1:50, BioGenex, Fremont, CA, USA) followed by Alexa Fluor 594-conjugated donkey anti-mouse IgG (1:200; Thermo Fisher Scientific, Warsaw, Poland), and GATA4, a PE marker—with rabbit polyclonal antibody (1:100; Santa Cruz Biotechnology, Dallas, TX, USA) followed by an Alexa-633-conjugated goat anti-rabbit IgG (1:200; Thermo Fisher Scientific, Warsaw, Poland). Embryos were incubated in a mix of primary antibodies overnight at 4°C, washed in PBS and 3% BSA, and then incubated in a mix of secondary antibodies for 2 h at room temperature. DNA was stained with chromomycin A3 (0.01 mg/ml in PBS; Merck, Poznań, Poland; 30 min at room temperature or overnight, 4°C). Embryos were analyzed on an inverted confocal microscope (510 LSM Meta, Zeiss, Jena, Germany). Cell numbers were calculated manually in Fiji software (Schindelin *et al.*, 2012).

### Outgrowth assay

E4.0 embryos (regardless of their developmental stage, i.e. both morulae and blastocysts) were plated individually in 96-well plates pre-coated with 0.2% gelatin (Merck, Poznań, Poland) and cultured for 4 days under standard conditions (37.5°C, 5% CO_2_). Wells were filled with ES medium without leukemia inhibitory factor (LIF). The ES medium contained knockout DMEM (Thermo Fisher Scientific, Warsaw, Poland) supplemented with 15% fetal bovine serum (Thermo Fisher Scientific, Warsaw, Poland), streptomycin (50 µg/ml, Thermo Fisher Scientific, Warsaw, Poland), penicillin (50 units/ml, Warsaw, Thermo Fisher Scientific, Poland), nonessential amino acids (0.1 mM, Thermo Fisher Scientific, Warsaw, Poland), L-glutamine (2 mM, Thermo Fisher Scientific, Warsaw, Poland), and β-mercaptoethanol (0.1 mM, Merck, Poznań, Poland). At the end of *in vitro* culture, outgrowths were imaged in transmitted light on a stereomicroscope and their areas were measured in Fiji software (Schindelin *et al.*, 2012).

### Embryo transfer

E4.0 embryos were transferred to pseudopregnant 8- to 12-week-old F1 females obtained by mating with vasectomized 6- to 12-month-old F1 males of proven sterility. Those with vaginal plugs were treated as pseudopregnant and were used as recipients in the embryo transfer procedure. The embryo transfer was conducted as described before (Tarkowski, 1959; Wassarman and DePamphilis, 1993; Nagy *et al.*, 2002). In short, females at 0.5 days postconception were anesthetized by i.p. injection of Nembutal (75 μg/g of body weight; Merck, Poznań, Poland) and analgesia was induced by s.c. injection of Tolfedine (4 μg/g of body weight; Vetoquinol, Gorzów Wielkopolski, Polska) and administration of lidocaine (0.1 ml of 1% (10 mg/ml) solution; Polfa Warszawa S.A., Warsaw, Poland) near the incision site. Next, E4.0 embryos (morulae and blastocysts, priority being given to blastocysts) were transferred unilaterally into the right oviduct of each female. The number of embryos transferred varied between recipients but on average, 11 embryos were transferred to a single recipient (Supplementary Table S1). The effectiveness of asynchronous transfers has been confirmed before (López-Cardona *et al.*, 2015). The pups were delivered naturally. The cages were checked for pups daily and the number of pups was counted.

### Fertility test

The 8-week-old mice obtained from OCM-scanned or control embryos were mated with naturally obtained F1 mice of the opposite sex and bred together for 3 months to follow their fertility over several pregnancies. The cages were checked for pups daily. The number of deliveries and number of pups were recorded.

### Statistical analysis

Stata/SE 18.0 software (StataCorp LLC, College Station, TX, USA) was used for statistical analysis. The datasets were tested for normal distribution with Shapiro–Wilk test. Statistical analysis involved chi-squared test, Fisher’s exact test, parametric two-tailed Student’s *t*-test, non-parametric Mann–Whitney *U*-test, Wilcoxon signed-rank test, and Spearman’s rank correlation. We also conducted univariate logistic and linear regression analyses. The differences between groups were considered statistically significant at *P* < 0.05. Values in the text show mean ± SD, while graphs display medians and quartiles.

## Results

### OCM is a reliable method of assessing the number of nuclei in compacted mouse embryos

First, we wished to verify whether OCM allows for a reliable assessment of the number of nuclei in embryos. To this end, compacted embryos (72 h post-hCG) obtained by *in vivo* fertilization (n = 30) were stained with DNA dye, Hoechst 33342, and then scanned by OCM and imaged in the fluorescence microscope. Then, the numbers of nuclei were calculated for each of the imaging methods, for each embryo, and compared (Fig. 1B and C). Statistical analysis showed that the numbers of nuclei obtained by both imaging methods for each embryo were indeed almost identical (*P* = 0.72, Wilcoxon signed-rank test, and ρ = 0.97, *P* < 0.0001 for Spearman’s rank correlation) (Fig. 1D and E). Therefore, OCM can be applied to assess the number of nuclei in compacted mouse embryos.

### The number of cells in compacted embryos is strongly associated with the ability of embryos to complete preimplantation development *in vitro*

**Figure 2.**
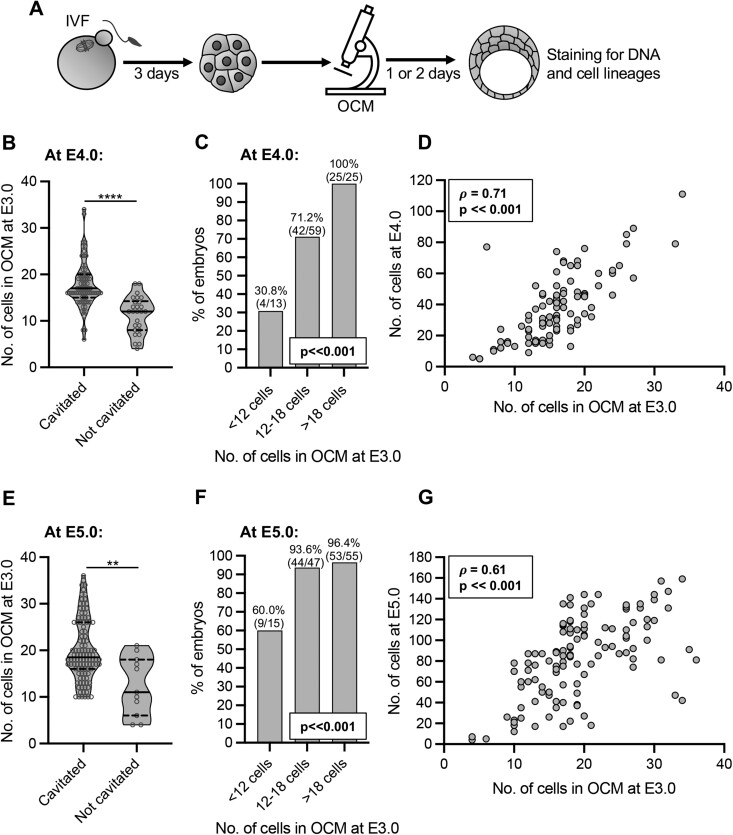
**Relation between the number of cells assessed by optical coherence microscopy in E3.0 mouse embryos and their preimplantation development**. (**A**) Scheme of the experimental design. (**B**) Relation between the number of cells assessed by optical coherence microscopy (OCM) in E3.0 (72 h after onset of insemination) embryos and their ability to cavitate by E4.0 (96 h after onset of insemination). Violin plots show distribution of the analyzed data; the solid black line indicates median, the dashed black lines—first and third quartile values; ****P ≪ 0.001. (**C**) Percentage of embryos reaching the blastocyst stage by E4.0 in relation to their cell number assessed by OCM at E3.0. (**D**) Spearman’s rank correlation between the numbers of cells in embryos at E3.0 and E4.0. (**E**) Relation between the number of cells assessed by OCM in E3.0 embryos and their ability to cavitate by E5.0 (120 h after onset of insemination). Violin plots show distribution of the analyzed data; the solid black line indicates median, the dashed black lines—first and third quartile values; **P < 0.01. (**F**) Percentage of embryos reaching the blastocyst stage by E5.0 in relation to their cell number assessed by OCM at E3.0. (**G**) Spearman’s rank correlation between the numbers of cells in embryos at E3.0 and E5.0. Ninety-seven embryos (71 that cavitated and 26 that did not) were analyzed in (B–D) and 117 embryos (106 that cavitated and 11 that did not) in (E–G).

Next, we investigated whether the number of nuclei in compacted embryos (a proxy for the cell number) assessed by OCM scanning reflects the embryo’s ability to cavitate, hatch, and form the first embryonic cell lineages, namely TE, EPI, and PE. E3.0 (i.e. 72 h post insemination) embryos obtained by IVF were imaged with OCM and then cultured individually for an additional 24 h (to E4.0) or 48 h (to E5.0), then fixed and stained for DNA (all nuclei), CDX2 (TE marker), and GATA4 (PE marker) (Fig. 2A). Cells negative for CDX2 and GATA4 staining were considered to belong to EPI. The experiment was conducted in four replicates (n = 9 mice) or six replicates (n = 12 mice) for embryos cultured for additional 24 or 48 h after OCM, respectively.

Embryos that did not form blastocoel at E4.0 (n = 26) and E5.0 (n = 11) had significantly fewer cells in OCM scans at E3.0 than embryos that cavitated (n = 71 and 106, respectively; *P* ≪ 0.001 and *P* < 0.01, Mann–Whitney *U*-test; Fig. 2B and E). This result was confirmed by univariate logistic regression analysis (Table 1). The analysis indicated that an increase in cell number at E3.0 by 1 led, on average, to a 44% and 27% increase in the likelihood of cavitation at E4.0 and E5.0, respectively. Importantly, we showed as well that embryos with a low number of cells at E3.0 (<12 cells) cavitated significantly less frequently than embryos with a higher number of cells (12–18 cells or >18 cells, *P* ≪ 0.001, chi-squared test; Fig. 2C and F).

**Table 1.** Univariate logistic regression analysis of the number of nuclei visualized by optical coherence microscopy at E3.0 in relation to mouse embryo ability to cavitate, hatch, and form an outgrowth.

Output parameters	No. of embryos	Odds ratio	95% CI		*P*-value
Cavitation at E4.0	97	1.44	1.22	1.70	<0.001
Cavitation at E5.0	117	1.27	1.10	1.47	0.001
Hatching at E5.0	106	1.18	1.09	1.28	<0.001
Formation of outgrowth^a^	115	1.37	1.20	1.55	<0.001
Formation of outgrowth with ICM	84	1.32	1.11	1.56	0.002

a Includes all outgrowths, with and without inner cell mass (ICM).

E3.0, E4.0, E5.0—72, 96, and 120 h after onset of insemination.

We also observed a significant correlation between the number of cells at E3.0 and the total cell number in embryos at E4.0 and E5.0 (Fig. 2D and G). This association was confirmed by univariate linear regression analysis (Table 2). Coefficient values indicated that if the number of cells at E3.0 embryos increases by 1, the number of cells in the resulting E4.0 embryos increases on average by 2.9 cells and in E5.0 embryos by 3.3 cells.

**Table 2.** Univariate linear regression analysis of the number of nuclei visualized with optical coherence microscopy at E3.0 in relation to the number of cells in the mouse embryos and the size of their outgrowths.

Output parameters	No. of embryos			Coefficient	95% CI		*P*-value	
**All embryos at E4.0**								
Total no. of cells	97			2.87	2.33	3.41	<0.001	
**Only blastocysts at E4.0**								
Total no. of cells	70			2.38	1.69	3.07	<0.001	
No. of TE cells	70			2.04	1.45	2.63	<0.001	
No. of ICM cells	70			0.33	0.12	0.54	0.002	
No. of EPI cells	70			0.20	0.01	0.39	0.039	
No. of PE cells	70			0.13	0.06	0.21	0.001	
**All embryos at E5.0**								
Total no. of cells	117			3.25	2.41	4.08	<0.001	
**Only blastocysts at E5.0**								
Total no. of cells	103			2.43	1.58	3.29	<0.001	
No. of TE cells	103			2.06	1.36	2.75	<0.001	
No. of ICM cells	103			0.38	0.13	0.62	0.003	
No. of EPI cells	103			0.15	0.02	0.28	0.025	
No. of PE cells	103			0.23	0.09	0.36	0.001	
**Outgrowths**								
Outgrowth area (μm^2^)		84	5693.1		3489.1	7897.1		<0.001

E3.0, E4.0, E5.0—72, 96, and 120 h after onset of insemination.

EPI: epiblast; ICM: inner cell mass; PE: primitive endoderm; TE: trophectoderm.

Then, we examined the association between the number of cells in E3.0 embryos and the quality of blastocysts obtained at E4.0 or E5.0, assessed as their ability to hatch and as numbers of cells they contained (total and in the first embryonic cell lineages) (Milewski *et al.*, 2018). Our analysis indicated that blastocysts that did not start hatching by E5.0 (n = 48) had significantly fewer cells in OCM scans at E3.0 than their hatching counterparts (n = 58; *P* ≪ 0.001, Mann–Whitney *U*-test; Supplementary Fig. S1A). This result was confirmed by univariate logistic regression analysis (Table 1), which indicated that an increase in the cell number at E3.0 by 1 led on average to an 18% increase in the likelihood of hatching at E5.0. Of note is that embryos with a low number of cells at E3.0 (<12 cells) initiated hatching by E5.0 significantly less frequently than embryos with a higher number of cells (12–18 cells or >18 cells, *P* < 0.05, Fisher’s exact test; Supplementary Fig. S1B).

Furthermore, the number of cells at E3.0 correlated with the total number of cells in E4.0 blastocysts and the number of cells in their TE, ICM (i.e. EPI and PE cells taken together), and PE. The number of cells at E3.0 correlated with the same parameters of E5.0 blastocysts and additionally with the number of their EPI cells (Table 3). Univariate linear regression analysis confirmed these relationships (Table 2). For example, coefficient values indicated that if the number of cells at E3.0 embryos increases by 1, the total number of cells in the resulting E4.0 blastocysts increases on average by 2.4 cells and the number of TE cells by 2.0, whereas in E5.0 embryos by 2.4 and 2.1 cells, respectively.

**Table 3.** Spearman correlation between the number of nuclei visualized by optical coherence microscopy at E3.0 and the quality of mouse blastocysts at E4.0 and E5.0.

Correlation between no. of nuclei in OCM at E3.0 and:	rho	*P*-value
**Blastocysts at E4.0**		
Total no. of cells	0.581	≪ 0.001
No. of TE cells	0.587	≪ 0.001
No. of ICM cells	0.248	< 0.05
No. of EPI cells	0.146	NS
No. of PE cells	0.527	≪ 0.001
**Blastocysts at E5.0**		
Total no. of cells	0.555	≪ 0.001
No. of TE cells	0.574	≪ 0.001
No. of ICM cells	0.346	< 0.001
No. of EPI cells	0.268	< 0.01
No. of PE cells	0.378	< 0.001

E3.0, E4.0, E5.0—72, 96, and 120 h after onset of insemination.

EPI: epiblast; ICM: inner cell mass; OCM: optical coherence microscopy; PE: primitive endoderm; TE: trophectoderm. NS: *P* > 0.05.

In summary, our results indicate that the number of cells in compacted E3.0 embryos, counted as the number of nuclei visualized by OCM, is tightly related to the embryo’s ability to cavitate, initiate hatching, and form the first embryonic cell lineages.

### The number of cells in compacted embryos is associated with the ability of embryos to implant *in vitro*

**Figure 3.**
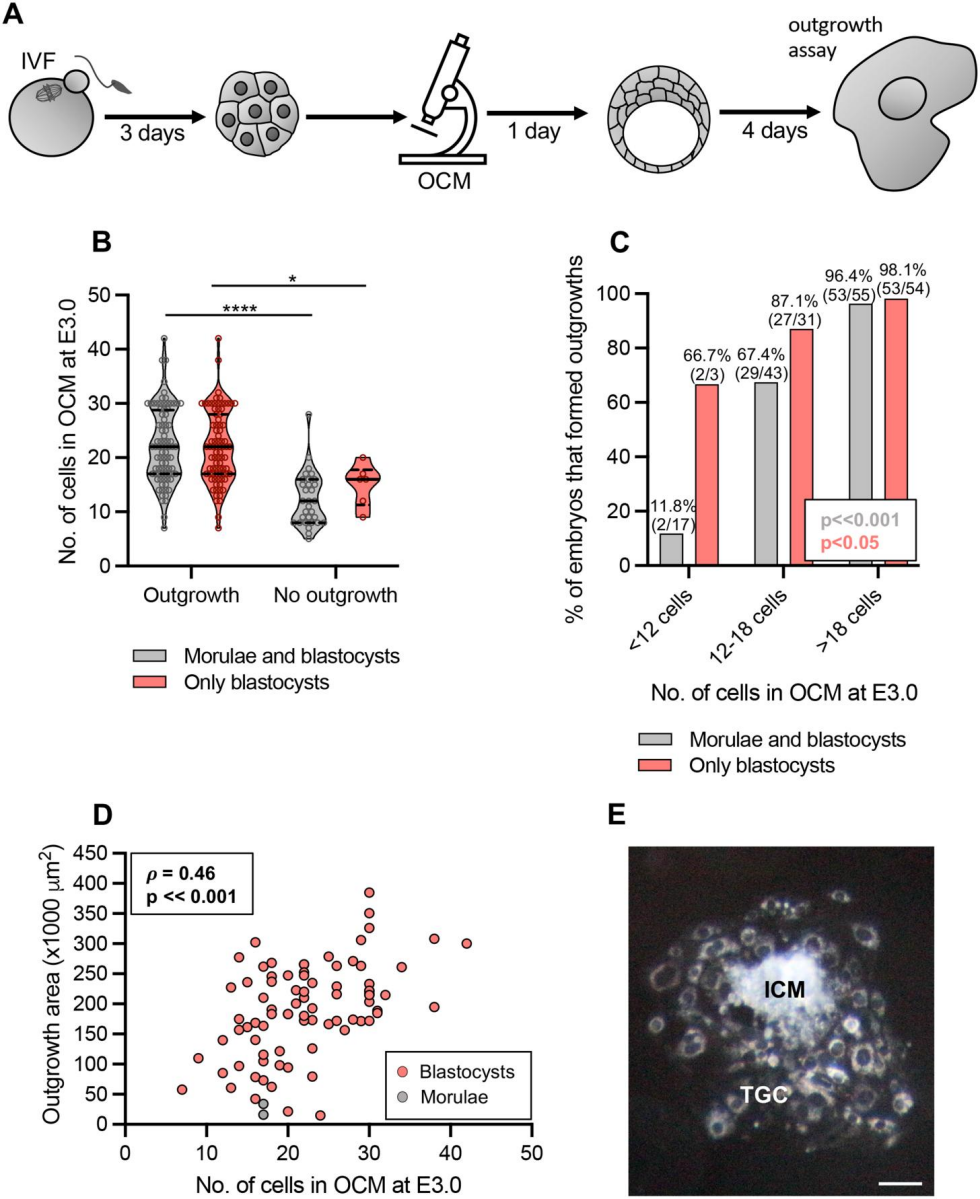
**Relation between the number of cells assessed by optical coherence microscopy in E3.0 mouse embryos and their ability to implant *in vitro***. (**A**) Scheme of the experimental design. (**B**) Relation between the number of cells assessed by optical coherence microscopy (OCM) in E3.0 (72 h after onset of insemination) embryos and their ability to form outgrowths. Violin plots show distribution of the analyzed data; the solid black line indicates median, the dashed black lines—first and third quartile values; *P < 0.05, ****P ≪ 0.001. (**C**) Percentage of embryos forming outgrowths in relation to their cell number assessed by OCM at E3.0. (**D**) Spearman’s rank correlation between the number of cells in embryos at E3.0 and the size of outgrowths formed by these embryos. (**E**) A representative image of a properly formed outgrowth. Scale bar 100 µm. (B–D) Embryos that were morulae or blastocysts on the day of plating for outgrowths (in gray) or only embryos that were blastocysts at that time (in red) were considered. One-hundred and fifteen embryos (84 that formed outgrowths and 31 that did not) were analyzed in (B and C) and 84 embryos in (D). ICM: inner cell mass; TGC: trophoblast giant cells.

Next, we wanted to examine whether the number of nuclei (i.e. cells) in E3.0 embryos assessed by OCM is associated with the embryo’s ability to implant. To this end, we imaged E3.0 embryos obtained by IVF with OCM, then cultured them individually for an additional 24 h and plated them for outgrowth (*in vitro* implantation assay) (Fig. 3A and E). As we wished to allow the embryonic cells to differentiate, our outgrowths were cultured in medium without LIF. The experiment was conducted in four replicates (n = 8 mice). Most OCM-scanned embryos managed to achieve the blastocysts stage at E4.0, i.e. before outgrowth plating (77%, 88/115), although some (23%, 27/115) were plated at the morula stage.

Embryos (morulae and blastocysts) that were able to form outgrowths within 4 days of culture (n = 84) had significantly more cells at E3.0 than those that did not implant *in vitro* (n = 31; *P* ≪ 0.001, Mann–Whitney *U*-test; Fig. 3B, gray plots). Importantly, only two embryos that were plated at the morula stage managed to form outgrowths. The same tendency in the E3.0 cell number was observed when only embryos that managed to reach the blastocyst stage before the outgrowth plating were considered (n = 82 and 6, respectively; *P* < 0.05, Mann–Whitney *U*-test; Fig. 3B, red plots). The result was additionally supported by univariate logistic regression analysis, which indicated that an increase in the number of cells in E3.0 embryos by 1 led to an average increase in a likelihood of outgrowth formation by 37% (Table 1). Importantly, embryos that had fewer cells at E3.0 (<12 cells) implanted *in vitro* less frequently than embryos that had more cells (12–18 cells and >18 cells; *P* ≪ 0.001, Fisher’s exact test, Fig. 3C, gray columns). This difference, although less pronounced, was also reported when only embryos that managed to reach the blastocyst stage before the outgrowth plating were considered (*P* < 0.05, Fisher’s exact test; Fig. 3C, red columns).

Additionally, we noticed that not all outgrowths were properly formed, i.e. displayed both ICMs and trophoblast cells (Supplementary Fig. S1C). Therefore, we tested whether their morphology was related to the number of cells in E3.0 embryos. We noticed that embryos (morulae and blastocysts) that formed outgrowths with ICMs (n = 72) had significantly more cells at E3.0 than those that created outgrowths without ICM (n = 12; *P* < 0.001, Mann–Whitney *U*-test; Supplementary Fig. S1D, gray plots). Again, this difference was also observed when only embryos that managed to reach the blastocyst stage before the outgrowth plating were considered (*P* < 0.001, Mann–Whitney *U*-test; Supplementary Fig. S1D, red plots). The result was confirmed by univariate logistic regression analysis, which indicated that an increase in the number of cells in E3.0 embryos by 1 led to an average increase in the likelihood of outgrowth with ICM by 32% (Table 1). Embryos that had more than 18 cells at E3.0 formed outgrowths with ICMs significantly more frequently than E3.0 embryos that had <18 cells (*P* < 0.001, chi-squared test; Supplementary Fig. S1E, gray columns). The same was true when only embryos that achieved the blastocyst stage before outgrowth plating were considered (*P* < 0.001, chi-squared test; Supplementary Fig. S1E, red columns).

Finally, we showed a significant correlation between the number of cells in E3.0 embryos and the outgrowth size (Fig. 3D). This observation was backed up by univariate linear regression analysis, which indicated that an increase in cell number in E3.0 embryos by 1 led to increasing the outgrowth size on average by over 5600 µm^2^ (Table 2).

In summary, we showed that the number of cells in compacted E3.0 embryos, counted as the number of nuclei visualized by OCM, may be tightly related to the ability of an embryo to implant. Importantly, our results indicate that the OCM-derived information on E3.0 embryos may help to differentiate between E4.0 blastocysts more and less able to implant.

### OCM does not hinder the developmental potential of the embryos

**Figure 4.**
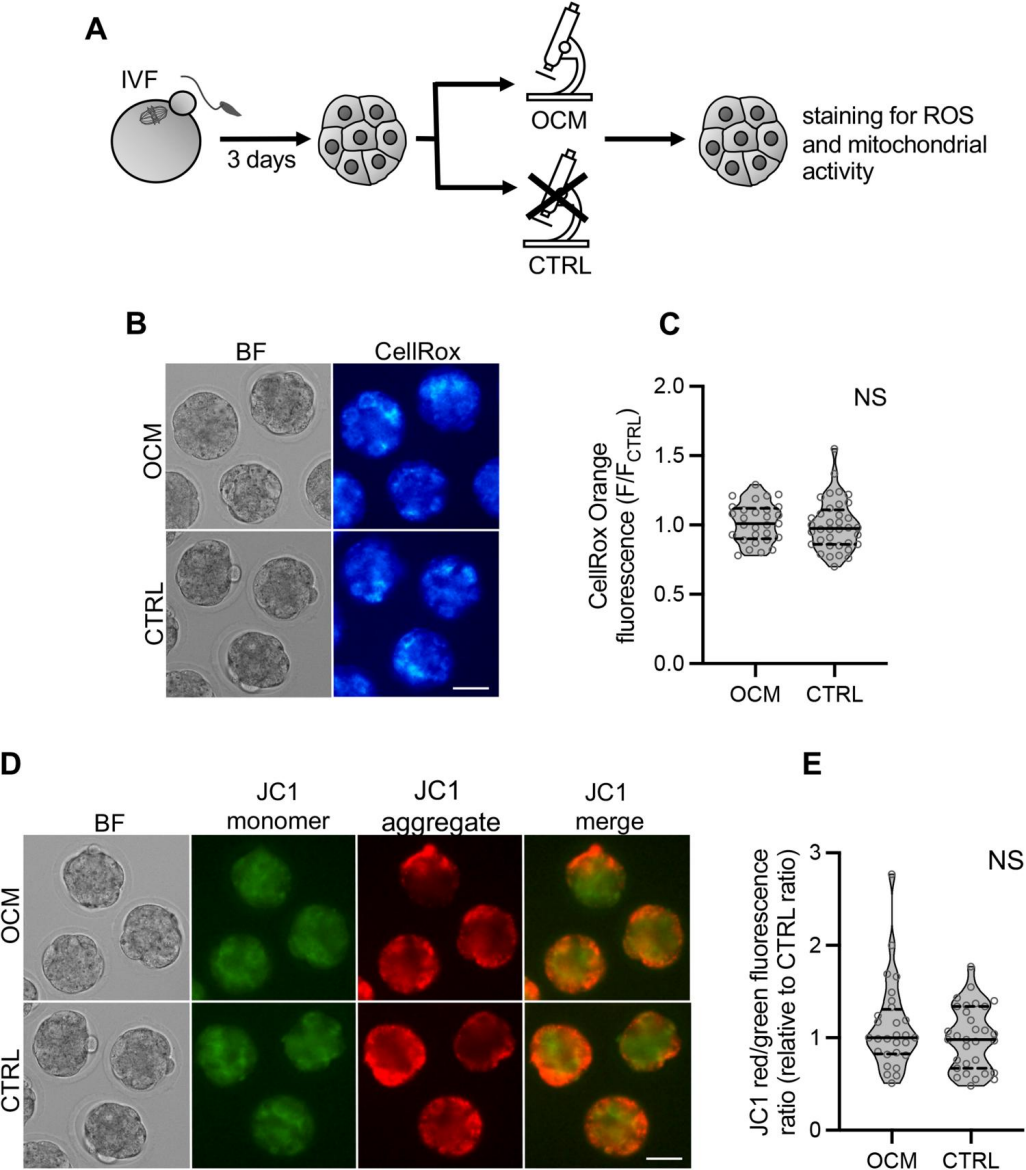
**Impact of optical coherence microscopy imaging on the amount of reactive oxygen species and mitochondrial activity in E3.0 mouse embryos**. (**A**) Scheme of the experimental design. (**B**) Representative images of embryos scanned with optical coherence microscopy (OCM) and control embryos labeled for reactive oxygen species with CellRox Orange dye. Scale bar 50 μm. (**C**) Relative intensity of CellRox Orange fluorescence in OCM-imaged (n = 29) and control (n = 36) embryos. (**D**) Representative images (single planes) of OCM-scanned and control oocytes labeled with JC-1, an indicator of mitochondrial activity. In mitochondria with high membrane potential, JC-1 forms aggregates emitting red fluorescence. In mitochondria with low membrane potential, JC-1 remains in the monomeric form emitting green fluorescence. Scale bar 50 μm. (**E**) Relative ratios of JC-1 red to green fluorescence intensities in OCM-imaged (n = 28) and control (n = 31) embryos. A higher ratio indicates more active mitochondria. (C and E) Violin plots show distribution of the analyzed data; the solid black line indicates median, the dashed black lines—first and third quartile values; NS: *P* > 0.05. BF: bright field; CTRL: the control group, embryos unscanned; OCM: the experimental group, embryos scanned with optical coherence microscopy; ROS: reactive oxygen species.

In the last set of experiments, we wished to test whether our OCM protocol is safe for embryos. As imaging is often linked to photoinduced oxidative stress (Pomeroy and Reed, 2012), we first investigated whether OCM scanning affected the amount of ROS and mitochondrial activity in compacted E3.0 embryos (Fig. 4A). To this end, OCM-scanned and control, unimaged embryos were incubated either with CellRox Orange, a fluorescent indicator of ROS, or with JC-1, a mitochondrial dye sensitive to mitochondrial membrane potential. The experiment was conducted in three replicates (n = 6 mice).

We found that the amount of ROS in OCM-scanned (n = 29) and unimaged embryos (n = 36) was the same (*P* > 0.05, Mann–Whitney *U*-test; Fig. 4B and C). Similarly, mitochondrial membrane potential was unchanged (*P* > 0.05, Mann–Whitney *U*-test; Fig. 4D and E) in OCM-scanned embryos (n = 28) as compared to their unimaged control counterparts (n = 31).

**Figure 5.**
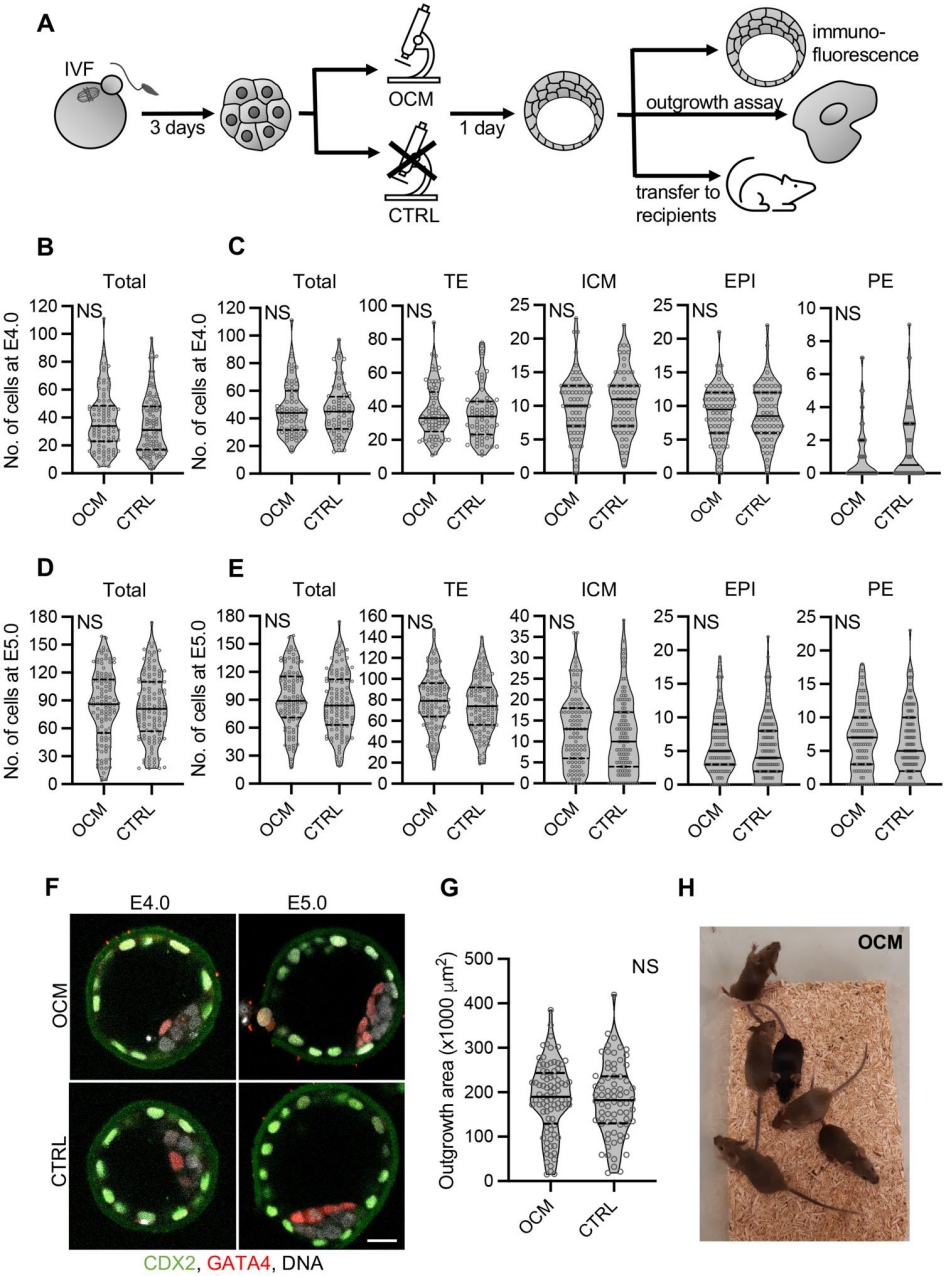
**Impact of optical coherence microscopy imaging on mouse embryo development**. (**A**) Scheme of the experimental design. (**B**) Total numbers of cells in control embryos or embryos scanned with optical coherence microscopy (OCM) at E3.0 (72 h after onset of insemination) and cultured to E4.0 (96 h after onset of insemination). (**C**) Numbers of cells (total, trophectoderm, inner cell mass, epiblast, and primitive endoderm) in control embryos or embryos scanned with OCM at E3.0 that cavitated by E4.0. (**D**) Total numbers of cells in control embryos or embryos scanned with OCM at E3.0 and cultured to E5.0 (120 h after onset of insemination). (**E**) Numbers of cells (total, trophectoderm, inner cell mass, epiblast, and primitive endoderm) in control embryos or embryos scanned with OCM at E3.0 that cavitated by E5.0. (**F**) Images of representative E4.0 and E5.0 embryos from OCM-scanned and control groups. CDX2, trophectoderm marker, in green, GATA4, primitive endoderm marker, in red, and DNA in white. Scale bar 20 µm. (**G**) Size of outgrowths formed by embryos subjected to OCM scanning or control. (**H**) Mice (1-month-old) born from embryos subjected to OCM scanning. (B–E and G) Violin plots show distribution of the analyzed data; the solid black line indicates median, the dashed black lines—first and third quartile values; NS: *P* > 0.05; 97 OCM-scanned and 103 control embryos were analyzed in (B), 70 and 64 in (C), 117 and 123 in (D), 103 and 115 in (E). Eighty-four and 72 outgrowths originating from OCM-scanned and control embryos, respectively, were analyzed in (G). CTRL: the control group, embryos unscanned; EPI: epiblast; ICM: inner cell mass; OCM: the experimental group, embryos scanned with optical coherence microscopy; PE: primitive endoderm; TE: trophectoderm.

Next, we compared the ability to complete pre- and postimplantation development by embryos that were or were not imaged by OCM at E3.0 (Fig. 5A). The experiment was conducted in 4–7 replicates (n = 8–14 mice), depending on the variant.

OCM-imaged and control, non-imaged embryos developed to the blastocyst stage at day E4.0 and E5.0 with the same frequency (*P* > 0.05, chi-squared test; Table 4) and had a similar number of cells (total and in the first embryonic cell lineages—TE, ICM, PE, and EPI, *P* > 0.05, Student’s *t*-test and Mann–Whitney *U*-test; Fig. 5B–F). Moreover, they implanted *in vitro* with the same frequency (*P* > 0.05, chi-squared test; Table 4) and their outgrowths were of similar size (*P* > 0.05, Student’s *t*-test; Fig. 5G). Most importantly, OCM imaging did not hinder an embryos’ ability to develop full term: the live birth rates were the same for OCM-scanned and control embryos (Fig. 5H, Table 4, Supplementary Table S1). We also compared the fertility of males and females obtained from OCM-scanned and control embryos by mating them with wild-type F1 mice. Both the number of deliveries and the mean number of pups per delivery were the same for OCM-scanned and control groups (*P* > 0.05, Mann–Whitney *U*-test; Table 5, Supplementary Table S2), which is promising, even if the number of animals compared in this part of the study was not sufficient to completely exclude the impact of OCM on the fertility of animals obtained from scanned embryos. Finally, mice obtained from OCM-scanned embryos did not display visible health defects (they were kept and observed in the animal facility for over 12 months).

**Table 4.** Cavitation, hatching, implantation *in vitro*, and live birth rates in scanned and control mouse embryos.

	OCM-scanned embryos	Control embryos
% of cavitation at E4.0	**73.2** (71/97)	**62.1** (64/103)
% of cavitation at E5.0	**90.6** (106/117)	**93.5** (115/123)
% of hatching at E5.0	**54.7** (58/106)	**48.7** (56/115)
% of outgrowth formation^a^	**73.0** (84/115)	**66.1** (72/109)
% of outgrowths with ICMs	**85.7** (72/84)	**88.9** (64/72)
% of live birth (per all embryos transferred)	**30.6** (33/108)	**28.7** (41/143)
% of live birth (per all blastocysts transferred)	**37.5** (33/88)	**33.3** (41/123)

a Includes all outgrowths, with and without inner cell mass (ICM).

OCM: optical coherence microscopy; E4.0, E5.0—96 and 120 h after onset of insemination.

**Table 5.** Fertility of mice derived from scanned and control embryos.

	Mice from OCM-scanned embryos		Mice from control embryos	
	Females	Males	Females	Males
No. of mice tested	8	9	7	6
No. of deliveries/3 months	3.4 ± 0.7	3.3 ± 0.7	3.7 ± 0.5	3.3 ± 0.8
No. of pups/delivery	8.5 ± 1.8	7.5 ± 3.0	9.0 ± 1.5	9.1 ± 0.6

OCM: optical coherence microscopy.

In summary, these results indicate that OCM scanning is safe for preimplantation mouse embryos and does not hinder their ability to complete full-term development.

## Discussion

In the present paper, we showed that the number of nuclei and, in consequence, the number of cells in compacted E3.0 embryos can be easily, safely, and reliably assessed by OCM—a feat that cannot be achieved with standard light microscopy methods. During compaction, blastomeres flatten against each other, losing their clear individual outlines and becoming difficult to distinguish (White *et al.*, 2016). OCM provides a solution to this problem, as it allows for visualization of nuclei in compacted embryos without fluorescent labelling of DNA and, consequently, estimating their cell number. It is particularly important as the timing of compaction relies, at least in part, on a mechanism independent of cellular divisions (Levy *et al.*, 1986; Lee *et al.*, 2001). This means that evaluation of embryo morphogenetic progress may not reflect real developmental potential. Indeed, it has been shown in humans that the number of blastomeres at the compaction initiation, and not only compaction timing, provides important information on embryo quality (Iwata *et al.*, 2014; Matot *et al.*, 2023). Therefore, cell count conducted at the compacted embryo stage presents an important update on the embryo status just prior to major developmental events, such as cavitation or implantation, and may be potentially more informative than analogical data recorded for earlier, noncompacted, embryonic stages.

It is important to note that although in mouse embryos multinucleated blastomeres are rare, in human embryos, this phenomenon occurs much more frequently (reviewed in Coticchio *et al.*, 2021a). Thus, multinucleation may potentially skew the result of OCM-based analysis of the cell number. However, it has been shown that multinucleation is most frequent at the earliest cleavage stages and tends to be self-corrected over time (Aguilar *et al.*, 2016; Balakier *et al.*, 2016; Coticchio *et al.*, 2021a; Sayed *et al.*, 2022), which suggests that at the morula stage, its prevalence may be already limited. Additionally, the potential error introduced by multinuclear blastomeres could be mitigated by combining OCM-based analysis with a standard morphological (e.g. number of nuclei per cell at earlier, noncompacted stages, percentage of fragmentation) or morphokinetic assessment (e.g. the time required to reach 2-, 4-, 5-, or 6-cell stage) that could indicate the risk of multinucleation (Royen, 2003; Ergin *et al.*, 2014; Aguilar *et al.*, 2016; Balakier *et al.*, 2016; Sayed *et al.*, 2022).

Noteworthily, our results clearly indicate that the number of nuclei at E3.0, as a proxy of cell number, correlates tightly with the developmental potential of embryos. We showed that it is associated with the quality of preimplantation development—the ability to reach a blastocyst stage, the number of cells at the end of the preimplantation period, and the ability to form the first embryonic cell lineages. This association between the number of cells at E3.0 and the ability to complete preimplantation development successfully has a strong biological rationale, as embryos with fewer cells were most likely dividing more slowly, which may suggest problems with either nuclear or cytoplasmic components. Slow divisions may be caused by a dysfunctional cytoskeleton, insufficient energy production owing to damaged mitochondria, or DNA damage activating cell-cycle checkpoints and halting the cell-cycle progression (reviewed in Ajduk and Zernicka-Goetz, 2013).

Our results show as well that the number of nuclei (cells) in E3.0 embryos correlates with the embryos’ ability to form outgrowths. Importantly, the association exists even if we consider only those embryos that were blastocysts at the time of the initiation of outgrowth culture, i.e. we exclude embryos that did not reach the blastocysts stage on time, so were potentially of lower developmental quality. It means that we can distinguish blastocysts that are less and more able to implant, at least *in vitro*. Outgrowths are a well-established model of implantation (Kim *et al.*, 2020). In our approach, we plated embryos on gelatin-coated plastic in medium devoid of LIF, as we wished to allow the embryonic cells to differentiate and, in the case of TE, to invade their milieu. Although, as for every model, an outgrowth assay is a simplified version of the *in vivo* process, some major embryonic mechanisms, such as cellular adhesion, differentiation, and spreading of trophoblast cells, are similar (Wang and Armant, 2002). Our data show that the higher number of cells in E3.0 embryos, the higher chance of forming an outgrowth and the higher the outgrowth size. Again, this seems to have a strong biological explanation: embryos (or blastocysts) with a higher number of cells tend to have a higher number of properly differentiated TE cells that can transform into the outgrowth trophoblast giant cells and facilitate implantation.

Interestingly, the correlation between E3.0 cell number and the total number of cells in embryos at later stages weakens with time: it is stronger for E4.0 than E5.0 embryos. Analogically, the correlation is stronger between the E3.0 cell number and the total cell numbers in E4.0 or E5.0 than with the outgrowth size. This tendency indicates that with time, other factors, unaccounted for in the OCM-based analysis, become increasingly important for embryo quality.

Last but not least, our data confirmed that the OCM protocol used in our experiments is safe for mouse embryos: it does not interfere with their pre- and postimplantation development. Moreover, the mice born out of OCM-scanned embryos were healthy and fertile. This accords with our previous data on OCM safety: we showed before that OCM imaging does not hinder the quality of mouse oocytes (Fluks *et al.*, 2022). However, it is important to note that our postimplantation embryo viability test had certain limitations. We used separate females to host OCM-scanned and control embryos, which means that they develop in different, although most likely very similar, oviductal and uterine environments. Additionally, the number of embryos transferred varied between recipients (although the mean number of embryos (and blastocysts) transferred per recipient in OCM-scanned and control groups were statistically the same), which could also affect conditions of the postimplantation development.

Although our study clearly indicates that OCM-based assessment of the nuclei (cell) number in compacted embryos may be a very valuable addition to the repertoire of the embryo quality assessment methods used in assisted reproduction protocols, it has certain limitations. Our experiments have been conducted on mouse embryos, which are a well-established model for mammalian (including human) developmental and reproductive biology. However, there are numerous differences between the embryonic development of mice and other species, also at the early (pre- and peri-implantation) stages, e.g. the prevalence of multinucleation mentioned above. Additionally, we did not follow thoroughly long-term effects of OCM scanning, such as epigenetic modifications, gene expression patterns, or health parameters in offspring, which may be an important issue worth addressing if the method is going to be applied in assisted reproduction. Therefore, our results should be verified for embryos of target species and the method itself may require some optimization before it can be applied in IVF practice.

## Supplementary Material

gaae012_Supplementary_Data

## Data Availability

The datasets generated and/or analyzed during the current study are available from the corresponding author at a reasonable request.

## References

[gaae012-B1] Aguilar J , Rubio I, Muñoz E, Pellicer A, Meseguer M. Study of nucleation status in the second cell cycle of human embryo and its impact on implantation rate. Fertil Steril 2016;106:291–299.e2. 27059510 10.1016/j.fertnstert.2016.03.036

[gaae012-B2] Ajduk A , Szkulmowski M. Light microscopy of mammalian gametes and embryos: methods and applications. Int J Dev Biol 2019;63:235–244. 31058300 10.1387/ijdb.180300aa

[gaae012-B3] Ajduk A , Zernicka-Goetz M. Quality control of embryo development. Mol Aspects Med 2013;34:903–918. 23563243 10.1016/j.mam.2013.03.001

[gaae012-B4] Anagnostopoulou C , Maldonado Rosas I, Singh N, Gugnani N, Chockalingham A, Singh K, Desai D, Darbandi M, Manoharan M, Darbandi S et al Oocyte quality and embryo selection strategies: a review for the embryologists, by the embryologists. Panminerva Med 2022;64:171–184. 35179016 10.23736/S0031-0808.22.04680-8

[gaae012-B5] Balakier H , Sojecki A, Motamedi G, Librach C. Impact of multinucleated blastomeres on embryo developmental competence, morphokinetics, and aneuploidy. Fertil Steril 2016;106:608–614.e2. 27206619 10.1016/j.fertnstert.2016.04.041

[gaae012-B7] Coticchio G , Barrie A, Lagalla C, Borini A, Fishel S, Griffin D, Campbell A. Plasticity of the human preimplantation embryo: developmental dogmas, variations on themes and self-correction. Hum Reprod Update 2021a;27:848–865. 34131722 10.1093/humupd/dmab016

[gaae012-B8] Coticchio G , Ezoe K, Lagalla C, Shimazaki K, Ohata K, Ninomiya M, Wakabayashi N, Okimura T, Uchiyama K, Kato K et al Perturbations of morphogenesis at the compaction stage affect blastocyst implantation and live birth rates. Hum Reprod 2021b;36:918–928. 33575789 10.1093/humrep/deab011

[gaae012-B10] Ebner T , Moser M, Shebl O, Sommergruber M, Gaiswinkler U, Tews G. Morphological analysis at compacting stage is a valuable prognostic tool for ICSI patients. Reprod Biomed Online 2009;18:61–66. 19146770 10.1016/s1472-6483(10)60425-7

[gaae012-B11] Ebner T , Moser M, Sommergruber M, Tews G. Selection based on morphological assessment of oocytes and embryos at different stages of preimplantation development: a review. Hum Reprod Update 2003;9:251–262. 12859046 10.1093/humupd/dmg021

[gaae012-B12] Ergin EG , Çalişkan E, Yalçinkaya E, Öztel Z, Çökelez K, Özay A, Özörnek HM. Frequency of embryo multinucleation detected by time-lapse system and its impact on pregnancy outcome. Fertil Steril 2014;102:1029–1033.e1. 25086787 10.1016/j.fertnstert.2014.06.030

[gaae012-B13] Fabozzi G , Alteri A, Rega E, Starita MF, Piscitelli C, Giannini P, Colicchia A. Morphological assessment on day 4 and its prognostic power in selecting viable embryos for transfer. Zygote 2016;24:477–484. 26350430 10.1017/S0967199415000404

[gaae012-B14] Fluks M , Tamborski S, Szkulmowski M, Ajduk A. Optical coherence microscopy allows for quality assessment of immature mouse oocytes. Reproduction 2022;164:83–95. 35900349 10.1530/REP-22-0178

[gaae012-B15] Fraser LR. Ca^2+^ is required for mouse sperm capacitation and fertilization *in vitro*. J Androl 1982;3:412–419.

[gaae012-B16] Fulton BP , Whittingham DG. Activation of mammalian oocytes by intracellular injection of calcium. Nature 1978;273:149–151. 565475 10.1038/273149a0

[gaae012-B17] Gallego RD , Remohí J, Meseguer M. Time-lapse imaging: the state of the art. Biol Reprod 2019;101:1146–1154. 30810735 10.1093/biolre/ioz035

[gaae012-B18] Hur C , Nanavaty V, Yao M, Desai N. The presence of partial compaction patterns is associated with lower rates of blastocyst formation, sub-optimal morphokinetic parameters and poorer morphologic grade. Reprod Biol Endocrinol 2023;21:12. 36709281 10.1186/s12958-023-01059-9 PMC9883889

[gaae012-B19] Ivec M , Kovacic B, Vlaisavljevic V. Prediction of human blastocyst development from morulas with delayed and/or incomplete compaction. Fertil Steril 2011;96:1473–1478.e2. 21982283 10.1016/j.fertnstert.2011.09.015

[gaae012-B20] Iwata K , Yumoto K, Sugishima M, Mizoguchi C, Kai Y, Iba Y, Mio Y. Analysis of compaction initiation in human embryos by using time-lapse cinematography. J Assist Reprod Genet 2014;31:421–426. 24610095 10.1007/s10815-014-0195-2 PMC3969466

[gaae012-B21] Karnowski K , Ajduk A, Wieloch B, Tamborski S, Krawiec K, Wojtkowski M, Szkulmowski M. Optical coherence microscopy as a novel, non-invasive method for the 4D live imaging of early mammalian embryos. Sci Rep 2017;7:4165. 28646146 10.1038/s41598-017-04220-8 PMC5482811

[gaae012-B23] Kim J , Lee J, Jun JH. Advantages of the outgrowth model for evaluating the implantation competence of blastocysts. Clin Exp Reprod Med 2020;47:85–93. 32521581 10.5653/cerm.2019.03216 PMC7315857

[gaae012-B24] Kovacs P. Embryo selection: the role of time-lapse monitoring. Reprod Biol Endocrinol 2014;12:124. 25510244 10.1186/1477-7827-12-124 PMC4290130

[gaae012-B26] Lee DR , Lee JE, Yoon HS, Roh SI, Kim MK. Compaction in preimplantation mouse embryos is regulated by a cytoplasmic regulatory factor that alters between 1- and 2-cell stages in a concentration-dependent manner. J Exp Zool 2001;290:61–71. 11429764 10.1002/jez.1036

[gaae012-B28] Levy JB , Johnson MH, Goodall H, Maro B. The timing of compaction: control of a major developmental transition in mouse early embryogenesis. Development 1986;95:213–237. 3794590

[gaae012-B29] Li HX , Xu XJ, Liu L. A new day 4 grading system to assess embryo quality in frozen embryo transfer cycles. Reprod Sci 2021;28:1333–1338. 33237518 10.1007/s43032-020-00389-y

[gaae012-B30] López-Cardona AP , Fernández-González R, Pérez-Crespo M, Alén F, Rodriguez de Fonseca F, Orio L, Gutierrez-Adan A. Effects of synchronous and asynchronous embryo transfer on postnatal development, adult health, and behavior in mice. Biol Reprod 2015;94:20. 26224009 10.1095/biolreprod.115.130385

[gaae012-B31] Masuda Y , Hasebe R, Kuromi Y, Kobayashi M, Urataki K, Hishinuma M, Ohbayashi T, Nishimura R. Three-dimensional live imaging of bovine preimplantation embryos: a new method for IVF embryo evaluation. Front Vet Sci 2021;8:639249. 33981741 10.3389/fvets.2021.639249 PMC8107228

[gaae012-B32] Matot R , Kalma Y, Rahav R, Azem F, Amir H, Ben-Yosef D. Cleavage stage at compaction—a good predictor for IVF outcome. Int J Gynaecol Obstet 2023;161:997–1003. 36495286 10.1002/ijgo.14619

[gaae012-B33] Mihajlović AI , Bruce AW. The first cell-fate decision of mouse preimplantation embryo development: integrating cell position and polarity. Open Biol 2017;7:170210. 29167310 10.1098/rsob.170210 PMC5717349

[gaae012-B34] Milewski R , Ajduk A. Time-lapse imaging of cleavage divisions in embryo quality assessment. Reproduction 2017;154:R37–R53. 28408705 10.1530/REP-17-0004

[gaae012-B35] Milewski R , Szpila M, Ajduk A. Dynamics of cytoplasm and cleavage divisions correlates with preimplantation embryo development. Reproduction 2018;155:1–14. 28993454 10.1530/REP-17-0230

[gaae012-B36] Montag M , Toth B, Strowitzki T. New approaches to embryo selection. Reprod Biomed Online 2013;27:539–546. 23933036 10.1016/j.rbmo.2013.05.013

[gaae012-B37] Moore EL , Wang S, Larina IV. Staging mouse preimplantation development in vivo using opticalcoherence microscopy. J Biophotonics 2019;12:e201800364. 30578614 10.1002/jbio.201800364 PMC6470020

[gaae012-B38] Nagy A , Gertsenstein M, Vintersten K, Behringer T. Manipulating the Mouse Embryo: A Laboratory Manual, 3rd edn. New York, NY: Cold Spring Harbor Laboratory Press, 2002.

[gaae012-B40] Płusa B , Piliszek A. Common principles of early mammalian embryo self-organisation. Development 2020;147:dev183079. 32699138 10.1242/dev.183079

[gaae012-B41] Pomeroy KO , Reed ML. The effect of light on embryos and embryo culture. J Reprod Stem Cell Biotechnol 2012;3:46–54.

[gaae012-B42] Raghunathan R , Singh M, Dickinson ME, Larin KV. Optical coherence tomography for embryonic imaging: a review. J Biomed Opt 2016;21:50902. 27228503 10.1117/1.JBO.21.5.050902 PMC4881290

[gaae012-B43] Royen EV. Multinucleation in cleavage stage embryos. Hum Reprod 2003;18:1062–1069. 12721185 10.1093/humrep/deg201

[gaae012-B44] Sayed S , Reigstad MM, Petersen BM, Schwennicke A, Hausken JW, Storeng R. Nucleation status of Day 2 pre-implantation embryos, acquired by time-lapse imaging during IVF, is associated with live birth. PLoS One 2022;17:e0274502. 36137104 10.1371/journal.pone.0274502 PMC9498959

[gaae012-B45] Schindelin J , Arganda-Carreras I, Frise E, Kaynig V, Longair M, Pietzsch T, Preibisch S, Rueden C, Saalfeld S, Schmid B et al Fiji: an open-source platform for biological-image analysis. Nat Methods 2012;9:676–682. 22743772 10.1038/nmeth.2019 PMC3855844

[gaae012-B46] Szkulmowski M , Wojtkowski M, Bajraszewski T, Gorczyńska I, Targowski P, Wasilewski W, Kowalczyk A, Radzewicz C. Quality improvement for high resolution in vivo images by spectral domain optical coherence tomography with supercontinuum source. Opt Commun 2005;246:569–578.

[gaae012-B47] Tarkowski AK. Experiments on the development of isolated blastomeres of mouse eggs. Nature 1959;184:1286–1287. 13836947 10.1038/1841286a0

[gaae012-B48] Tsai NC , Su YT, Lin YJ, Chiang HJ, Huang FJ, Kung FT, Lan KC. Developmental potential of surplus morulas with delayed and/or incomplete compaction after freezing-thawing procedures. Reprod Biol Endocrinol 2019;17:87. 31666062 10.1186/s12958-019-0535-2 PMC6821030

[gaae012-B49] Wang J , Armant DR. Integrin-mediated adhesion and signaling during blastocyst implantation. Cells Tissues Organs 2002;172:190–201. 12476048 10.1159/000066970

[gaae012-B50] Wassarman PM , DePamphilis ML. Guide to techniques in mouse development. Methods in Enzymology. Vol. 225, San Diego, CA: Academic Press, 1993.

[gaae012-B51] White MD , Bissiere S, Alvarez YD, Plachta N. Mouse embryo compaction. In: DePamphilis ML (ed). Mammalian Preimplantation Development. Cambridge, MA: Elsevier, 2016, 235–258. 10.1016/bs.ctdb.2016.04.005 27475854

[gaae012-B52] Xiao J , Wang B, Lu G, Zhu Z, Huang Y. Imaging of oocyte development using ultrahigh-resolution full-field optical coherence tomography. Appl Opt 2012;51:3650–3654. 22695605 10.1364/AO.51.003650

[gaae012-B53] Zheng J , Chen T, Wang C, Tian N, Zhao F, Huo T, Zhang N, Chen D, Ma W, Sun JL et al Label-free subcellular 3D live imaging of preimplantation mouse embryos with full-field optical coherence tomography. J Biomed Opt 2012;17:070503. 22894459 10.1117/1.JBO.17.7.070503

[gaae012-B54] Zheng JG , Huo T, Tian N, Chen T, Wang C, Zhang N, Zhao F, Lu D, Chen D, Ma W et al Noninvasive three-dimensional live imaging methodology for the spindles at meiosis and mitosis. J Biomed Opt 2013;18:50505. 23698317 10.1117/1.JBO.18.5.050505

