# Supplementary material for: The number of nuclei in compacted embryos, assessed by optical coherence microscopy, is a non-invasive and robust marker of mouse embryo quality
Source: Mol Hum Reprod. 2024 Feb 24;30(4):gaae012. doi: 10.1093/molehr/gaae012 (PMC10978378; doi:10.1093/molehr/gaae012)
Supplement: gaae012_Supplementary_Data [file gaae012_supplementary_data.pdf]

Supplementary Information

for

The number of nuclei in compacted embryos, assessed by optical coherence microscopy, is a non-invasive and robust marker of mouse embryo quality

by

Aleksandra Sobkowiak, Monika Fluks, Ewa Kosyl, Robert Milewski, Marcin Szpila, Szymon Tamborski, Maciej Szkulmowski, Anna Ajduk

Contents:

1. **Supplementary Fig. S1.** Relation between the number of cells assessed by optical coherence microscopy in E3.0 embryos and their subsequent ability to hatch and differentiate into properly formed outgrowths.
2. **Supplementary Table S1.** Details of embryo transfers for embryos imaged with optical coherence microscopy (OCM) and control embryos (CTRL).
3. **Supplementary Table S2.** Details of breeding experiments testing fertility of offspring derived from embryos imaged with optical coherence microscopy (OCM) and control embryos (CTRL).

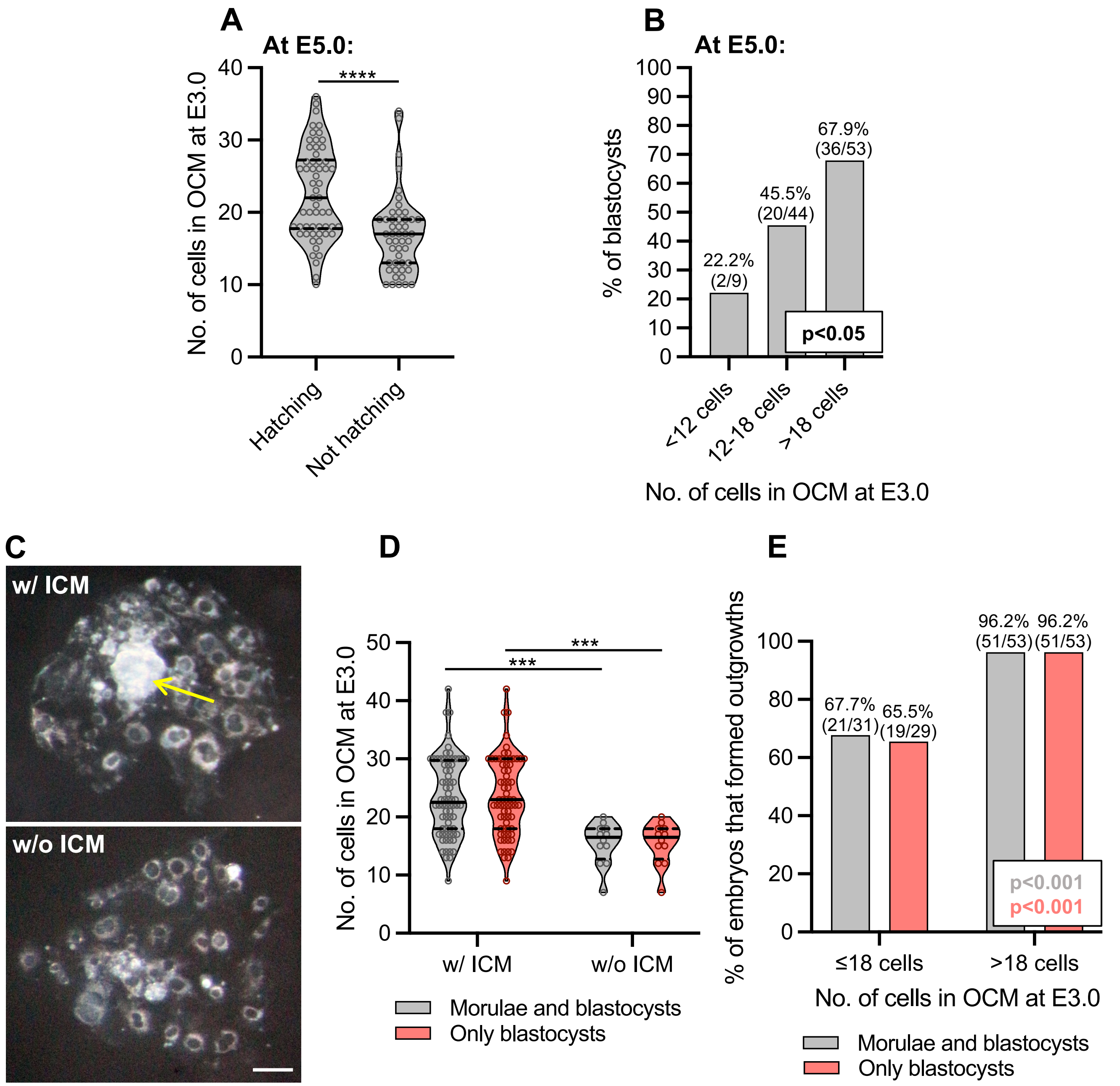

Supplementary Fig. S1

Relation between the number of cells assessed by optical coherence microscopy in E3.0 embryos and their subsequent ability to hatch and differentiate into properly formed outgrowths.

(A) Relation between the number of cells assessed by optical coherence microscopy (OCM) in E3.0 (72 hrs after onset of insemination) embryos and their ability to hatch by E5.0 (120 hrs after onset of insemination). Violin plots show distribution of the analysed data; the solid black line indicates median, the dashed black lines – 1st and 3rd quartile values; ****$p << 0.001$. **(B)** Percentage of blastocysts hatching by E5.0 in relation to their cell number assessed by OCM at E3.0. **(C)** Representative images of outgrowths with and without inner cell mass (marked with an arrow). Scale bar 100 µm. **(D)** Relation between the number of cells assessed by OCM in E3.0 embryos and their ability to form outgrowths with inner cell masses. Violin plots showing distribution of the analysed data; the solid black line indicates median, the dashed black lines – 1st and 3rd quartile values; ***$p < 0.001$. **(E)** Percentage of embryos forming outgrowths with inner cell masses in relation to their cell number assessed by OCM at E3.0. **(D, E)** Embryos that were morulae or blastocysts on the day of plating for outgrowths (in grey) or only embryos that were blastocysts at that time (in red) were considered. 106 embryos (58 hatching and 48 not hatching) were analysed in **(A, B)** and 84 outgrowths (72 with inner cell masses and 12 without) in **(D, E)**. ICM – inner cell mass.

Supplementary Table S1.
Details of embryo transfers for embryos imaged with optical coherence microscopy (OCM) and control embryos (CTRL).

Recipient no.	No. of E4.0 embryos transferred (morulae and blastocysts)	No. of blastocysts transferred	% of blastocysts (among all transferred embryos)	No. of pups	Live birth rate (% of all transferred embryos)	Live birth rate (% of transferred blastocysts)
OCM_1.1	9	8	88.9	5	55.6	62.5
OCM_1.2	11	6	54.5	4	36.4	66.7
OCM_2.1	12	12	100.0	0	0.0	0.0
OCM_2.2	12	12	100.0	7	58.3	58.3
OCM_3.1	10	4	40.0	0	0.0	0.0
OCM_3.2	8	5	62.5	2	25.0	40.0
OCM_4.1	5	2	40.0	1	20.0	50.0
OCM_5.1	14	12	85.7	1	7.1	8.3
OCM_5.2	15	15	100.0	6	40.0	40.0
OCM_6.1	12	12	100.0	7	58.3	58.3
MEAN	10.8	8.8	77.2	3.3	30.1	38.4
SD	2.9	4.4	25.4	2.8	23.2	26.2
CTRL_1.1	9	5	55.6	0	0.0	0.0
CTRL_1.2	10	6	60.0	5	50.0	83.3
CTRL_2.1	11	11	100.0	1	9.1	9.1
CTRL_2.2	13	13	100.0	4	30.8	30.8
CTRL_3.1	17	3	76.5	5	29.4	38.5
CTRL_4.1	6	1	16.7	0	0.0	0.0
CTRL_5.1	14	13	92.9	0	0.0	0.0
CTRL_5.2	15	13	86.7	0	0.0	0.0
CTRL_6.1	12	12	100.0	6	50.0	50.0
CTRL_6.2	12	12	100.0	7	58.3	58.3
CTRL_7.1	12	12	100.0	6	50.0	50.0
CTRL_7.2	12	12	100.0	7	58.3	58.3
MEAN	11.9	10.3	82.4	3.4	28.0	31.5
SD	2.8	4.0	26.2	3.0	24.9	29.1

E4.0 – 96 hrs after onset of insemination

Supplementary Table S2

Details of breeding experiments testing fertility of offspring derived from embryos imaged with optical coherence microscopy (OCM) and control embryos (CTRL).

Tested female no.	No. of pups				
	1st delivery	2nd delivery	3rd delivery	4th delivery	Total
OCM_Fe1	5	7	3	-	15
OCM_Fe2	7	10	8	-	25
OCM_Fe3	8	7	11	-	26
OCM_Fe4	8	9	10	10	37
OCM_Fe5	8	11	10	14	43
OCM_Fe6	10	9	8	12	39
OCM_Fe7	9	9	8	11	37
OCM_Fe8	7	7	-	-	14
MEAN	7.8	8.6	8.3	11.8	29.5
SD	1.5	1.5	2.6	1.7	11.1

Tested male no.	No. of pups				
	1st delivery	2nd delivery	3rd delivery	4th delivery	Total
OCM_Ma1	9	8	10	-	27
OCM_Ma2	7	9	5	7	28
OCM_Ma3	10	3	9	-	22
OCM_Ma4	9	9	9	7	34
OCM_Ma5	9	10	-	-	19
OCM_Ma6	10	12	7	-	29
OCM_Ma7	2	1	1	-	4
OCM_Ma8	11	11	10	11	43
OCM_Ma9	9	2	1	5	17
MEAN	8.4	7.2	6.5	7.5	24.8
SD	2.7	4.1	3.8	2.5	11.1

Tested female no.	No. of pups				
	1st delivery	2nd delivery	3rd delivery	4th delivery	Total
CTRL_Fe1	8	10	10	10	38
CTRL_Fe1	10	9	10	9	38
CTRL_Fe2	7	7	10	6	30
CTRL_Fe4	9	11	10	-	30
CTRL_Fe5	10	2	9	-	21
CTRL_Fe6	18	9	6	10	33
CTRL_Fe7	10	13	10	12	45
MEAN	8.9	8.7	9.3	9.4	33.6
SD	1.2	3.5	1.5	2.2	7.7

Tested male no.	No. of pups				
	1st delivery	2nd delivery	3rd delivery	4th delivery	Total
CTRL_Ma1	9	10	10	9	38
CTRL_Ma2	8	9	12	-	29
CTRL_Ma3	10	9	-	-	19
CTRL_Ma4	8	9	9	8	34
OCM_Ma5	8	7	10	-	25
OCM_Ma6	8	7	10	11	36
MEAN	8.5	8.5	10.2	9.3	30.2
SD	0.8	1.2	1.1	1.5	7.3
